# Expression of RABEX-5 and its clinical significance in prostate cancer

**DOI:** 10.1186/1756-9966-33-31

**Published:** 2014-04-09

**Authors:** Hongtuan Zhang, Shang Cheng, Andi Wang, Hui Ma, Bing Yao, Can Qi, Ranlu Liu, Shiyong Qi, Yong Xu

**Affiliations:** 1 Department of Urology, National Key Clinical Specialty of Urology, Second Hospital of Tianjin Medical University, Tianjin Key Institute of Urology, Tianjin medical university, Tianjin, China; 2 Department of gynaecology and obstetrics, Second Hospital of Tianjin Medical University, Tianjin medical university, Tianjin, China

## Abstract

**Background:**

While recent research has shown that expression of RABEX-5 in breast cancer and colorectal cancer has a crucial impact on tumor development, there is little information regarding RABEX-5 expression in prostate cancer. This study investigated the expression of RABEX-5 in prostate cancer by real time quantitative polymerase chain reaction and evaluated its association with clinicopathological variables, including prostate cancer patient prognosis.

**Methods:**

A total of 180 patients with primary prostate cancer treated by radical prostatectomy were enrolled. Real time quantitative polymerase chain reaction was utilized to investigate mRNA expression level of RABEX-5 in 180 paired prostate cancer/adjacent non-cancerous tissues. RABEX-5 mRNA expression was divided into high expression group and low expression group and correlations between RABEX-5 mRNA and clinicopathological factors were then evaluated. Kaplan-Meier plots and Cox proportional hazards regression model were used to analyze the association between RABEX-5 mRNA expression and prognosis of patients with prostate cancer.

**Results:**

Our study showed that RABEX-5 mRNA was significantly upregulated in prostate cancer tissues. The data indicated that high expression of RABEX-5 mRNA was significantly associated with lymph node metastasis (P = 0.001), clinical stage (P = 0.004), biochemical recurrence (P = 0.009), preoperative prostate-specific antigen (P < 0.001), and Gleason score (P < 0.001). High RABEX-5 mRNA expression was a significant predictor of poor biochemical recurrence free survival and overall survival both in univariate and multivariate analysis.

**Conclusion:**

This is to our knowledge the first report investigating tumor RABEX-5 mRNA expression level in prostate cancer. We have shown that high RABEX-5 mRNA expression is a strong predictor of poor prognosis in prostate cancer patients treated by radical prostatectomy, and multivariate analysis confirmed RABEX-5 mRNA as an independent prognostic factor.

## Background

Prostate cancer is the second most common cancer in men and account for approximately 28,170 deaths in 2012 [1]. Even when prostate cancer is apparently confined to the prostate, it encompasses a broad spectrum of prostate cancer, some of which are characterized by extremely indolent behavior and others by very poor outcome [2,3]. Recent efforts have focused on developing effective biomarkers that provide clinicians with the improved ability to identify clinically significant prostate cancer and aid in treatment decision. Therefore, an important clinical question is how aggressively to treat prostate cancer patients. Prostate cancer patients and clinicians are in need of more accurate biomarkers to predict the prognosis of prostate cancer, especially for intermediate grade tumors. Few biomarkers have been reported that reliably predict treatment failure. New prognostic biomarkers are therefore required.

Rab-type small GTPases are conserved membrane trafficking proteins in all eukaryotes, and they mediate various steps in membrane trafficking, including vesicle movement along cytoskeletons, vesicle docking to specific membranes, vesicle budding, and vesicle fusion [4,5]. Rabs function as a molecular switch by cycling between two nucleotide-bound states, a GDP-bound inactive state (“OFF” state) and a GTP-bound active state (“ON” state). Rabs are activated by specific guanine nucleotide exchange factors, which promote the release of GDP from Rab and binding of GTP to Rab, and the activated Rabs are then inactivated by GTPase-activating proteins or spontaneously inactivated by their intrinsic GTPase activity [6], either of which terminates the cycle [6,7]. Therefore, the identification and characterization of these Rab regulators, especially of GEFs, is crucial to understanding the spatiotemporal regulation of Rab GTPase activation.

The small GTPase RAB-5, which is found at the plasma membrane and early endosomes, is a master regulator of early endocytic trafficking [8]. Like other small GTPases, RAB-5 is activated by an exchange of bound GDP with GTP, which is catalyzed by a family of guanine-nucleotideexchange factors. RABEX-5 was identified as an interactor of Rabaptin-5 and was found to possess GEF activity toward RAB-5 and related GTPases. Likewise, both Rabaptin-5 and RABEX-5 are essential for RAB-5-driven endosome fusion in vitro [9].

Aberrant RABEX-5 expression may result in obstruction of the RAB-5-mediated endocytic vesicle fusion process, thereby causing defects in phagocytosis. The results showed that RABEX-5 was overexpressed in colorectal cancer and breast cancer [10,11]. The data indicated that RABEX-5 may act as an oncogene that is involved in the formation and development of malignant tumors and might influence tumor biological behavior. However, the role and mechanism of action of RABEX-5 in prostate cancer have not yet been studied. In present study, we first analyzed the expression of RABEX-5 in prostate cancer tissue by real time quantitative polymerase chain reaction. Subsequently, the association between RABEX-5 and prostate cancer clinicopathological factors was evaluated. Additionally, we assessed the influence of RABEX-5 mRNA expression on the biochemical recurrence free survival and overall survival of patients with prostate cancer.

### Tissue specimens

A total of 180 human prostate cancer and paired adjacent noncancerous tissues were obtained from the second hospital of Tianjin medical university, which underwent radical prostatectomy at this hospital between 1999 and 2010 [12-14]. Written informed consent was obtained from all prostate cancer patients and this study was approved by the research ethics committee of Tianjin medical university (TMUhMEC2013011). This investigation conformed to the principles outlined in the Declaration of Helsinki. Demographic and clinicopathological data of prostate cancer patients were collected from medical records. None of the prostate cancer patients received androgen deprivation treatment, chemotherapy, or radiation therapy prior to radical prostatectomy. The tissue samples were snapfrozen in liquid nitrogen and stored at -80°C until used. The histopathology of each specimen was reviewed on the HE-stained tissue section to confirm diagnosis and tumor content at least 70% of prostate cancer cells in the tissue samples. The following biochemical and clinicopathological parameters were recorded: biochemical relapse, preoperative serum prostate-specific antigen, clinical stage, lymph node status, angiolymphatic invasion status, Gleason score, margin status, and seminal vesicle invasion status. The time to biochemical recurrence was defined as the period between radical prostatectomy and the measurement of two successive values of serum prostate-specific antigen level ≥ 0.2 ng/ml.

### Quantitative real-time polymerase chain reaction

Total RNA was isolated from the 180 pairs of prostate cancer tissue and adjacent noncancerous tissues using TRIZOL reagent (Invitrogen). RNA was reverse-transcribed using SuperScript First Strand cDNA System (Invitrogen) according to the manufacturer’s instructions. The RABEX-5 sense primer was 5′-TTGGACAGATGGAATTGCAA-3′, and the antisense primer was 5′-GTTGCAGTGGTGGAGGAAGT-3′. For the β-actin gene, the sense primer was 5′-ATAGCACAGCCTGGATAGCAACGTAC-3′, and the antisense primer was 5′-CACCTTCTACAATGAGCTGCGTGTG-3′. Quantitative real-time polymerase chain reaction was conducted using SYBR Green polymerase chain reaction master mix (Applied Biosystems) in a total volume of 20 μl on the 7900HT fast Real-time polymerase chain reaction system (Applied Biosystems) as follows: 50°C for 2 minutes, 95°C for 15 minutes, 40 cycles of 95°C for 15 seconds, and 60°C for 60 seconds. A dissociation procedure was performed to generate a melting curve for confirmation of amplification specificity. β-actin was used as the reference gene. The relative levels of gene expression were represented as ΔCt = Ctgene- Ctreference, and the fold change of gene expression was calculated by the 2^-ΔΔCt^ Method. Experiments were repeated in triplicate.

### Statistical analysis

Statistical analysis was performed using SPSS version 17.0. Quantitative real-time polymerase chain reaction data were analyzed using Student’s t-test and expressed as mean ± SD. The correlation between RABEX-5 mRNA expression and the clinicopathological parameters was assessed by Chi-square test. Kaplan-Meier and log-rank tests were used when calculating the statistical significances of the overall survival rate and biochemical recurrence free survival rate, while COX regression analysis was used for the univariate and multivariate analysis. Multivariate survival analysis was performed on all parameters that were found to be significant on univariate analysis. Differences were considered statistically significant when P < 0.05.

## Results

### RABEX-5 mRNA expression is up-regulated in prostate cancer tissues compared to adjacent noncancerous tissues

**Figure 1:**
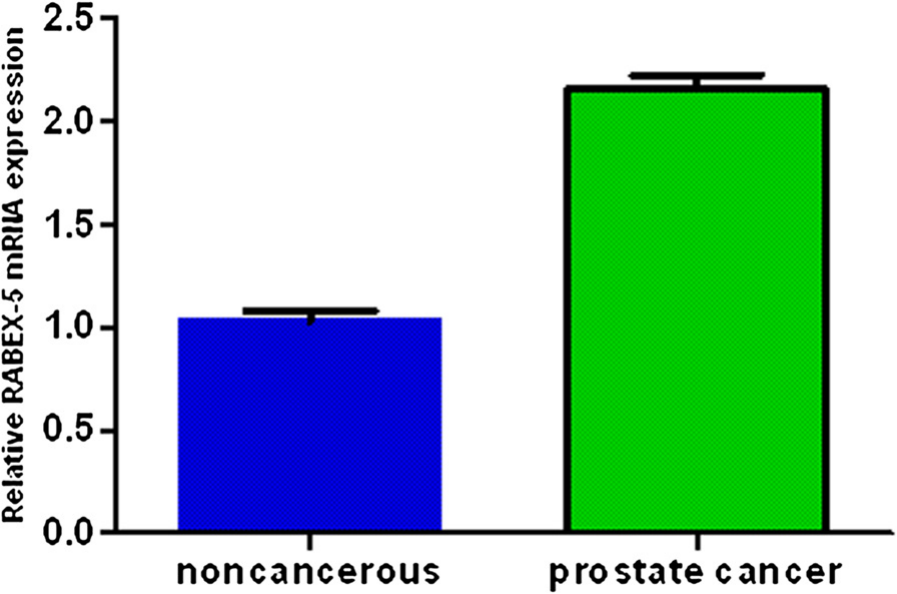
**Identification of upregulated RABEX-5 mRNA expression in prostate cancer tissues compared with its adjacent non-cancerous tissues by real time quantitative polymerase chain reaction.** The data reveal that RABEX-5 mRNA levels in the prostate cancer tissues were significantly higher than those in the adjacent non-cancerous tissues (P < 0.05).

Abnormally high RABEX-5 expression has been implicated in colorectal cancer and breast cancer, but the pathological function of RABEX-5 in prostate cancer has not been well defined. Therefore, quantitative real-time polymerase chain reaction analysis was performed on paired samples of prostate cancer tissue and noncancerous tissue adjacent to the cancer lesion isolated from the same patient. We determined the levels of RABEX-5 transcript in samples from prostate cancer and adjacent noncancerous tissues using quantitative real-time polymerase chain reaction. Our data reveal that RABEX-5 mRNA levels in the prostate cancer tissues were significantly higher than those in the adjacent non-cancerous tissues (Figure 1).

### Relationship between RABEX-5 mRNA expression and prostate cancer patients’ clinicopathological variables

The annotation of 180 prostate cancer patients includes clinical outcomes, and in particular survival and biochemical recurrence data, so we cross-checked these data with RABEX-5 mRNA expression levels. The 180 prostate cancer samples were subdivided into two groups with respectively low or high amounts of RABEX-5 mRNA. These groups were stratified by the median value. In our prostate cancer cohort, the relationship between the expression of RABEX-5 mRNA and patient clinical and pathological characteristics was shown in Table 1. High expression of RABEX-5 mRNA was found to significantly correlate with lymph node metastasis (P = 0.001), clinical stage (P = 0.004), preoperative prostate-specific antigen (P < 0.001), biochemical recurrence (P = 0.009), and Gleason score (P < 0.001). No significant difference in RABEX-5 mRNA expression was observed with age, surgical margin status, seminal vesicle invasion, and angiolymphatic invasion (P > 0.05).

**Table 1:** Main characteristics of studies included in this meta-analysis

		**RABEX-5 mRNA expression**			
**Variable**	**Group**	**High**	**Low**	**Total**	**P value**
Age					0.052
	<70	55 (56.7%)	42 (43.3%)	97	
	≥70	35 (42.2%)	48 (57.8%)	83	
Lymph node metastasis					0.001
	Absence	75 (46.0%)	88(54.0%)	163	
	Presence	15 (88.2%)	2 (11.8%)	17	
Surgical margin status					0.578
	Absence	82 (49.4%)	84 (50.6%)	166	
	Presence	8 (57.1%)	6 (42.9%)	14	
Seminal vesicle invasion					0.851
	Absence	73 (50.3%)	72 (49.7%)	145	
	Presence	17 (48.6%)	18 (51.4%)	35	
Clinical stage					0.004
	T1	42 (40.8%)	61 (59.2%)	103	
	T2/T3	48 (62.3%)	29 (37.7%)	77	
Preoperative PSA					< 0.001
	<4	1 (20%)	4 (80%)	5	
	4-10	20 (31.3%)	44 (68.7%)	64	
	>10	69 (62.2%)	42 (37.9%)	111	
Gleason score					
	<7	29 (29.3%)	70 (70.7%)	99	<0.001
	7	22 (64.7%)	18 (35.3%)	34	
	>7	39 (83.0%)	8 (17.0%)	47	
Angiolymphatic invasion					0.346
	Absence	75 (51.7%)	70 (48.3%)	145	
	Presence	15(42.9%)	20 (57.1%)	35	
Biochemical recurrence					0.009
	Absence	56 (43.8%)	72 (56.2%)	128	
	Presence	34 (65.4%)	18 (34.6%)	52	

### Relationship between clinicopathological variables, RABEX-5 mRNA expression, and biochemical recurrence free survival

**Figure 2:**
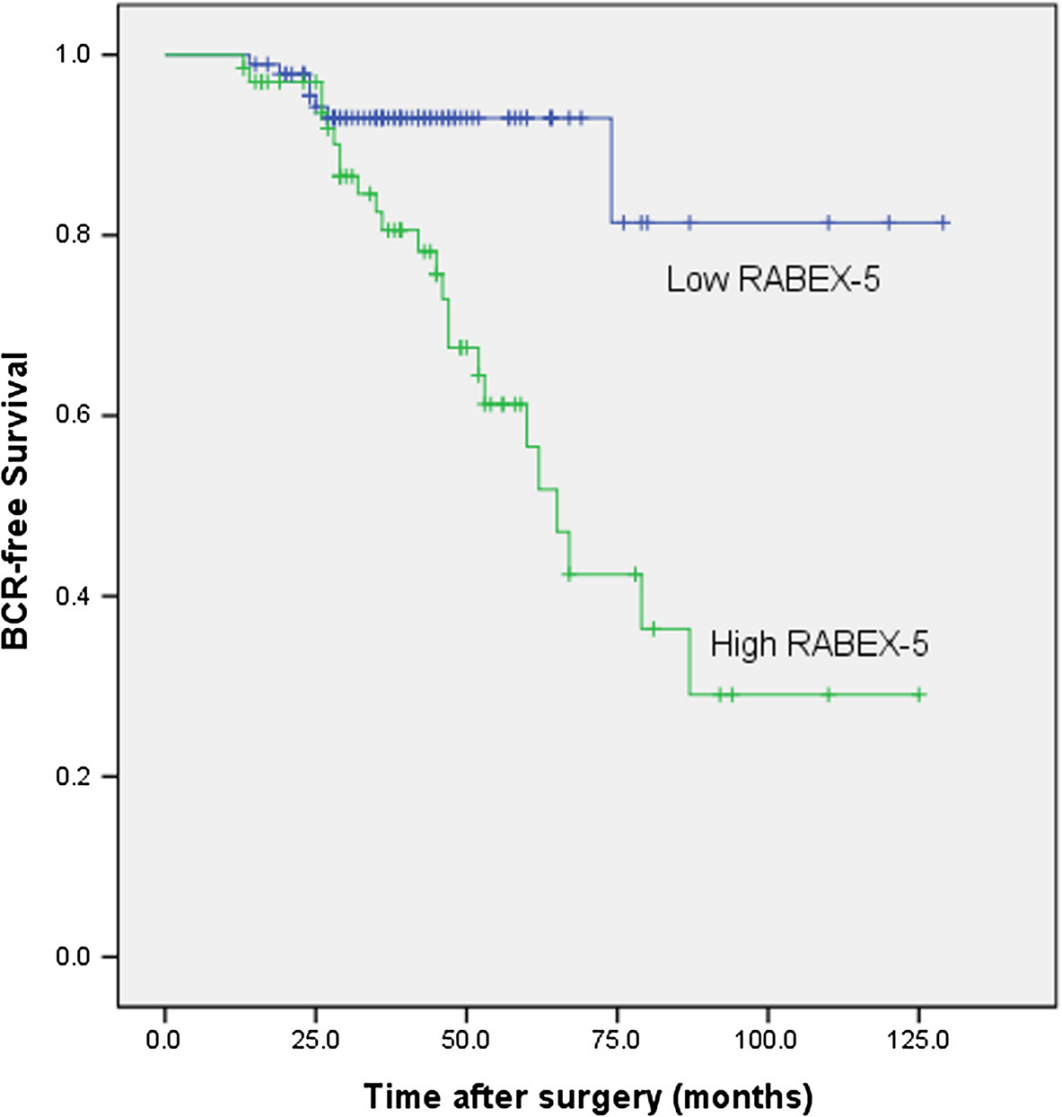
**Associations between RABEX-5 mRNA expression and biochemical recurrence free time after radical prostatectomy in patients with prostate cancer.** Patients with high RABEX-5 mRNA expression showed significantly shorter biochemical recurrence free survival than those with low RABEX-5 mRNA expression (P < 0.001, log-rank test).

In univariate survival analyses, cumulative survival curves were calculated according to the Kaplan-Meier method. Differences in survival times were assessed using the log rank test. First, to confirm the representativeness of the prostate cancer in present study, we analyzed established prognostic predictors of prostate cancer patient survival. Kaplan-Meier analysis demonstrated a significant impact of well-known clinicopathological prognostic parameters, such as seminal vesicle invasion, and Gleason score (P < 0.05, Table 2). Assessment of biochemical recurrence-free survival in total prostate cancer revealed that the high expression level of RABEX-5 mRNA was correlated with adverse biochemical recurrence free survival of prostate cancer patients (Figure 2). Since variables observed to have a prognostic influence by univariate analysis may covariate, the expression of RABEX-5 mRNA and those clinicalopathological parameters that were significant in univariate analysis were further examined in multivariate analysis. The results showed that the high expression of RABEX-5 mRNA was an independent prognostic factor for biochemical recurrence-free survival (relative risk: 1.642, 95% CI: 1.154-2.337, P = 0.006, Table 2). With regard to other parameters, Gleason score or seminal vesicle invasion status was shown to be an independent prognostic factor for biochemical recurrence-free survival.

**Table 2:** Prognostic value of RABEX-5 mRNA expression for the biochemical recurrence free survival in univariate and multivariate analyses by Cox regression

	**Univariate analysis**			**Multivariate analysis**		
**Covariant**	**Exp (B)**	**95% CI**	**P value**	**Exp (B)**	**95% CI**	**P value**
RABEX-5 mRNA expression	1.716	1.207-2.439	0.003	1.642	1.154-2.337	0.006
Gleason score	1.703	1.280-2.265	<0.001	1.674	1.259-2.225	<0.001
Seminal vesicle invasion	1.505	1.132-2.003	0.005	1.443	1.084-1.920	0.012
Preoperative PSA	1.241	0.705-2.188	0.454			
Angiolymphatic invasion	1.084	0.814-1.443	0.580			
Surgical margin status	1.017	0.709-1.459	0.925			
PCa Stage	1.090	0.921-1.291	0.316			
Lymph node metastasis	1.140	0.850-1.528	0.381			
Age	1.068	0.804-1.419	0.650			

### Relationship between clinicopathological variables, RABEX-5 mRNA expression, and overall survival

In terms of overall survival, patients with high RABEX-5 mRNA expression had a poorer overall survival than patients with low RABEX-5 mRNA expression. Prostate cancer patients with high RABEX-5 mRNA expression had shorter overall survival. Multivariate analysis indicated that high amount of RABEX-5 mRNA was associated with poorer overall survival, independently of classical clinical, biological and pathological features such as Gleason score, preoperative prostate-specific antigen, and prostate cancer stage (Table 3). The detailed data between RABEX-5 mRNA expression and overall survival are shown in Table 3.

**Table 3:** Prognostic value of RABEX-5 mRNA expression for the overall survival in univariate and multivariate analyses by Cox regression

	**Univariate analysis**			**Multivariate analysis**		
**Covariant**	**Exp (B)**	**95% CI**	**P value**	**Exp (B)**	**95% CI**	**P value**
RABEX-5 mRNA expression	1.629	1.038-2.555	0.034	1.751	1.098-2.792	0.019
Gleason score	2.526	1.788-3.568	<0.001	1.953	1.370-2.784	<0.001
Preoperative PSA	2.034	1.338-23.092	0.001	2.025	1.313-3.123	0.001
PCa Stage	4.131	2.888-5.911	<0.001	4.094	2.773-6.043	<0.001
Age	1.282	0.917-1.792	0.146			
Angiolymphatic invasion	1.373	0.813-2.319	0.235			
Surgical margin status	1.101	0.703-1.724	0.674			
Lymph node metastasis	1.044	0.746-1.462	0.800			
Seminal vesicle invasion	1.358	0.956-1.928	0.087			

## Discussion

Prostate cancer is the most frequently diagnosed malignant disease in men and the second leading cause of cancer deaths in the United States [1]. Prostate cancer poses a major public health problem in the United States and worldwide [1,12-14]. The treatment of prostate cancer with radical prostatectomy, which may be combined with chemotherapy, hormone therapy or radiation therapy, is curative in many patients with prostate cancer. However, most prostate cancer patients eventually relapse with castration-resistant prostate cancer and develop metastatic disease, which has a poor prognosis because no effective treatments are currently available [15,16]. Although prostate-specific antigen screening has become very common in the clinic, this marker lacks specificity [17]. Up to 25% patients with prostate cancer have prostate-specific antigen levels < 4.0 ng/ml, and elevated prostate-specific antigen levels can also result from benign prostatic disease [18]. A substantial proportion of screen-detected prostate cancers may have been overdiagnosed and subsequently overtreated, while others may not have been detected and treated early enough. The predictive value of conventional clinicopathological parameters for powerful prognosticators, such as pathological tumor stage and lymph node metastatic disease, remains limited [19,20]. Widespread overtreatment has greatly increased the social burden and poor quality of life. Despite the generally good prognosis for early stage prostate cancer patients, many affected individuals still die as a result of metastasis and recurrence, which is the major cause for most cancer-related deaths. Therefore, the identification of reliable biomarkers for identifying prostate cancer and predicting recurrence is critical for early diagnosis and prognostic evaluation, and for therapeutic molecular targets of prostate cancers [21,22]. Therefore, it is urgent to seek and refine prognostic variable, which is gained from pretreatment variables and prostate cancer biopsy specimens in particular [19].

Abnormally high RABEX-5 expression has been implicated in breast cancer and colorectal cancer, but the function of RABEX-5 in prostate cancer has not been well studied. To date, an association between RABEX-5 expression and prostate cancer has not been reported. Therefore, reverse transcription polymerase chain reaction analysis was performed on paired samples of prostate cancer tissue and noncancerous tissue adjacent to the cancer lesion isolated from the same patient. Our data showed that there is an elevation in RABEX-5 mRNA expression in prostate cancer tissues compared to adjacent noncancerous tissues. We next investigated the associations between abnormal RABEX-5 mRNA expression and clinicopathological factors. High expression of RABEX-5 mRNA was found to significantly correlate with lymph node metastasis, clinical stage, preoperative prostate-specific antigen, biochemical recurrence, and Gleason score. In contrast, there were no significant correlations between abnormal RABEX-5 mRNA expression and age, surgical margin status, seminal vesicle invasion, and angiolymphatic invasion. This is the first study to elucidate the clinicopathological significance of RABEX-5 mRNA expression in patients with prostate cancer.

In the present study we also have investigated the prognostic impact of RABEX-5 mRNA in a previously described cohort of 180 surgically resected prostate cancer patients [12-14]. To confirm the representativeness of the prostate cancer in present study, we analyzed established prognostic predictors of prostate cancer patient survival. The data showed a significant impact of well-known clinical pathological prognostic parameters, such as seminal vesicle invasion, and Gleason score. Assessment of biochemical recurrence free survival in prostate cancer revealed that the high expression level of RABEX-5 mRNA was correlated with adverse biochemical recurrence free survival of prostate cancer patients. Since variables observed to have a prognostic influence by univariate analysis may covariate, the expression of RABEX-5 mRNA and those clinicalopathological parameters that were significant in univariate analysis were further examined in multivariate analysis. Multivariate analysis revealed that RABEX-5 mRNA expression was an independent predictor of biochemical recurrence free survival. Our data demonstrate a marked increase in RABEX-5 mRNA expression in tumors compared to noncancerous tissue, with a significant and independent relationship between high RABEX-5 mRNA expressing tumors and reduced postoperative overall survival. It seems convincing that the high RABEX-5 mRNA expression conferred a very unfavorable prognosis in our study cohort. The high expression of RABEX-5 mRNA was a significant indicator for predicting poor outcome after radical prostatectomy. Therefore, a high RABEX-5 mRNA expression may play an important role on the growth of prostate cancer. This is the first study that demonstrates RABEX-5 mRNA to be an independent prognosticator in prostate cancer with high RABEX-5 mRNA expression indicating poor outcome. The finding that patients with high RABEX-5 mRNA expressing tumors have worse biochemical recurrence free and overall survival than patients with low RABEX-5 mRNA expressing tumors indicates that RABEX-5 mRNA has the potential to be used as a useful prognostic biomarker in prostate cancer. Consequently, RABEX-5 mRNA expression, if validated in future studies, could be used for selection of prostate cancer patients for adjuvant treatment following radical prostatectomy. Overall, our data show that high RABEX-5 mRNA expression profile correlates with poor prognosis in prostate cancer.

## Conclusions

In conclusions, RABEX-5 was found to be overexpressed at the mRNA level in prostate cancer samples examined compared to adjacent non-cancerous tissues from the same patient. Our current work demonstrates that RABEX-5 mRNA expression levels are associated with lymph node metastasis, clinical stage, preoperative prostate-specific antigen, biochemical recurrence, and Gleason score. RABEX-5 may play an important role in prostate cancer development. Our study has laid a foundation for future investigations to further explore the potential of RABEX-5 mRNA as a diagnostic marker for monitoring biochemical recurrence and as an effective therapeutic target for preventing and treating prostate cancer.

### Consent

Written informed consent was obtained from the patient for publication of this report and any accompanying images.

## Abbreviations

PSA: Prostate-specific antigen; BCR: Biochemical recurrence; CI: Confidence interval.

## Competing interests

The authors declare that they have no competing interests.

## Authors’ contributions

ZH, CS, MH, QC and XY conceived and designed the study, performed the experiments and wrote the paper. ZH, CS, WA, YB, and XY contributed to the writing and to the critical reading of the paper. ZH, MH, LR, WA, and QS performed patient collection and clinical data interpretation. ZH, CS, MH, YB, and QC participated performed the statistical analysis. All authors read and approved the final manuscript.
